# Emerging trends in gait recognition based on deep learning: a survey

**DOI:** 10.7717/peerj-cs.2158

**Published:** 2024-07-10

**Authors:** Vaishnavi Munusamy, Sudha Senthilkumar

**Affiliations:** School of Computer Science and Engineering, Vellore Institute of Technology, Vellore, Tamilnadu, India

**Keywords:** Deep learning, Convolutional neural network, Gait recognition, Person identification, Mask R-CNN, GEI

## Abstract

Gait recognition, a biometric identification method, has garnered significant attention due to its unique attributes, including non-invasiveness, long-distance capture, and resistance to impersonation. Gait recognition has undergone a revolution driven by the remarkable capacity of deep learning to extract complicated features from data. An overview of the current developments in deep learning-based gait identification methods is provided in this work. We explore and analyze the development of gait recognition and highlight its uses in forensics, security, and criminal investigations. The article delves into the challenges associated with gait recognition, such as variations in walking conditions, viewing angles, and clothing as well. We discuss about the effectiveness of deep neural networks in addressing these challenges by providing a comprehensive analysis of state-of-the-art architectures, including convolutional neural networks (CNNs), recurrent neural networks (RNNs), and attention mechanisms. Diverse neural network-based gait recognition models, such as Gate Controlled and Shared Attention ICDNet (GA-ICDNet), Multi-Scale Temporal Feature Extractor (MSTFE), GaitNet, and various CNN-based approaches, demonstrate impressive accuracy across different walking conditions, showcasing the effectiveness of these models in capturing unique gait patterns. GaitNet achieved an exceptional identification accuracy of 99.7%, whereas GA-ICDNet showed high precision with an equal error rate of 0.67% in verification tasks. GaitGraph (ResGCN+2D CNN) achieved rank-1 accuracies ranging from 66.3% to 87.7%, whereas a Fully Connected Network with Koopman Operator achieved an average rank-1 accuracy of 74.7% for OU-MVLP across various conditions. However, GCPFP (GCN with Graph Convolution-Based Part Feature Polling) utilizing graph convolutional network (GCN) and GaitSet achieves the lowest average rank-1 accuracy of 62.4% for CASIA-B, while MFINet (Multiple Factor Inference Network) exhibits the lowest accuracy range of 11.72% to 19.32% under clothing variation conditions on CASIA-B. In addition to an across-the-board analysis of recent breakthroughs in gait recognition, the scope for potential future research direction is also assessed.

## Introduction

Measuring the biological characteristics of an individual based on their unique physical or behavioral traits is a biometric. Throughout history, people have used various non-automated biometric methods (Chaudhari, Pawar & Deore, 2013; Connor & Ross, 2018; Cutting & Kozlowski, 1977). The earliest known reference to such methods dates back to prehistoric times, with hand ridge patterns discovered in Nova Scotia. Fingerprint recognition is among the oldest biometric methods, with records of its use dating back to 6000 B.C. by ancient civilizations, such as the Assyrians, Babylonians, Japanese, and Chinese. The study of fingerprints was formalized in the 19^th^ century, leading to classification systems. Anthropologist Alphonse Bertillon developed body measurement methods for identification, followed by the development of automated fingerprint systems in the late 20^th^ century. Other modalities, like iris and facial recognition, have contributed to the biometric evolution. By the mid-20^th^ century, advancements in signature and retinal scanning had emerged. Biometric industry organizations, such as IBA and IBIA, further propelled the growth of biometrics. Over the 20^th^ and 21^st^ centuries, biometrics has expanded across sectors, including law enforcement, commercial, and governmental applications.

Modern biometrics like gait recognition use analysis of distinctive walking patterns of individuals to identify them (Cai et al., 2021; Aung & Pluempitiwiriyawej, 2020; Ding et al., 2022; Jin et al., 2022). Unlike other biometric methods like face recognition, fingerprint recognition, and iris scanning, gait recognition operates from a distance and requires no direct interaction with the person being identified (Su, Zhao & Li, 2021; Zhang et al., 2022; Mu et al., 2020; Ding et al., 2022). Research has shown that even from poor-quality gait demonstrations, individuals can be recognized, highlighting the distinctiveness of gait as an identity marker. There are numerous applications of this technology, including person identification, criminal investigation, social security and surveillance, law enforcement, video monitoring authentication, healthcare, airport security, access control, pedestrian traffic monitoring in smart cities, sports, fitness tracking, and human-computer interaction (Liu et al., 2021; Lin et al., 2021). Its unique advantages include long-distance capture, non-invasiveness, and resistance to imitation, making it a reliable and secure identification method (Aung & Pluempitiwiriyawej, 2020).

In an era of growing identity theft concerns, individuals can employ strategies to evade traditional biometric systems, such as covering their faces or wearing gloves, masking one’s gait proves to be more challenging. Gait recognition technology offers a promising solution to address these concerns and a non-intrusive means of identifying individuals. Researchers have been actively working on improving gait recognition techniques over the past three decades, and their potential applications in crime investigation, forensic identification, and enhancing traditional biometric identification systems, making it a valuable and increasingly relevant field of study.

In this article, the fundamentals section describes the basic methods, advantages, gaps, and challenges of GAIT recognition technology, along with its utility in criminal investigations. The survey methodology elaborates on the strategy adopted to conduct the survey, followed by the steps involved in recognizing human gait using deep learning techniques. The literature review focuses on recent advancements in person identification by employing various gait recognition techniques. The technical details of the different datasets used in gait recognition are discussed along with their respective frequencies in gait analysis. The outcomes of various techniques, as reported in recent research, are appraised in the experimental results & analysis section. The conclusion outlines the substantial advances in recent years and directions for future research on gait recognition.

This study reviews recent advancements in gait recognition and examine the technical aspects and effectiveness of authentication techniques using walking patterns. The main contribution of this article:

- To provide readers with an in-depth theoretical understanding of gait recognition, tracing its roots in biometric recognition and outlining the most widely used tools in recent years for extracting gait features and architectures that address related constraints.
- To provide an illustrative, classified, and annotated list of publicly accessible datasets for gait recognition.
- To illustrate how gait recognition performs better in person identification tasks than face recognition.
- To provide a thorough analysis of the latest developments in gait recognition for person identification in the early 2020s, including a comparative review of experimental analyses.
- Discuss and highlight the advantages, limitations, methods, datasets, and significant results in the field, while also offering suggestions for future research directions.

## Fundamentals

This section enumerates the prerequisites of Gait Recognition Technology, highlighting its advantages, such as long-distance capture, non-cooperation requirement, and difficulty to imitate, making it a reliable identification method. Furthermore, the key applications of Gait Technology, including criminal investigations, healthcare, sports, biomechanics research, security, and robotics are examined.

### Advantages of gait recognition technology

When compared to other biometric identification systems Gait identification system has several advantages.

Long-distance capture: Gait recognition technology can accurately capture walking pattern of an individual from a distance, making it suitable for surveillance and large-scale video analysis (Su, Zhao & Li, 2021; Zhang et al., 2022; Mu et al., 2020; Han et al., 2022; Jin et al., 2022).

Non-cooperation requirement: Unlike other biometric recognition technologies that require active participation from the individual, gait recognition can be applied unobtrusively, without the cooperation of the person (Su, Zhao & Li, 2021; Zhang et al., 2022; Mu et al., 2020).

Difficulty in imitation: Gait recognition is difficult to fake or manipulate, making it a reliable means of identification (Cai et al., 2021; Aung & Pluempitiwiriyawej, 2020). Additionally, gait recognition has proven useful in crime investigation, forensic identification, and social security applications (Liu et al., 2021; Lin et al., 2021).

### Usefulness of gait recognition technology

The utilization of gait recognition technology has transformed a number of industries, including robotics, sports, law enforcement, and healthcare. This modern technology has unmatched potential for improving forensic investigations, reviving cold case inquiries, and validating testimonies and alibis. Gait analysis is essential for fall detection, injury prevention, and rehabilitation monitoring in medical settings. It also helps athletes and sportspeople perform better and avoid injuries. Its applications include wearable technology, biometric identification, human-computer interfaces, vehicle safety, and robotics, which makes it a multipurpose tool with enormous promise in a variety of fields. This in-depth study explored the various ways in which gait recognition technology is reshaping industries and advancing human endeavors.

### Cross-checking alibis and testimonies (Liu et al., 2021; Lin et al., 2021)

Utilizing spatiotemporal methods, gait recognition serves as a crucial tool for verifying or contradicting alibis and testimonies. Surveillance footage that captures unique gait patterns provides evidence of regarding the presence of an individual at a crime scene. By comparing these patterns with claimed alibis or witness descriptions, it verifies or contradicts the presence of individuals at specific locations and times. This objective evidence enhances the reliability of alibi verification and witness testimonies, strengthens the investigative process and contributes to the accuracy and fairness of legal proceedings.

### Criminal profiling (Liu et al., 2021; Lin et al., 2021)

Gait analysis in criminal profiling harnesses the unique walking styles of individuals, akin to fingerprints or DNA, to create suspect profiles. This process involves scrutinizing factors like stride length, speed, posture, and foot placement. By scrutinizing surveillance footage, law enforcement can extract valuable information on the gait patterns of a suspect, aiding in narrowing down the potential leads. In cases where traditional identification methods are inconclusive, gait analysis offers an additional means of pinpointing suspects by comparing observed patterns with databases. This technique not only facilitates the identification of potential suspects, but also aids in prioritizing investigative resources more effectively, increasing the likelihood of apprehending perpetrators.

### Forensic evidence (Liu et al., 2021; Lin et al., 2021)

Gait recognition technology enhances forensic investigations by analyzing distinctive walking patterns of individuals captured in surveillance footage. By correlating these patterns with specific locations and times, the presence or movement of individuals during critical events can be confirmed. These empirical data reinforce other case materials, such as DNA analysis or fingerprinting, by corroborating timelines and establishing connections between individuals and crime scenes. Moreover, gait recognition fills the gaps in traditional forensic evidence, particularly in cases of obscured visual identification. Overall, it strengthens the integrity of legal proceedings and contributes to more robust outcomes in the criminal justice system.

### Investigation of unsolved cases (Liu et al., 2021; Lin et al., 2021)

Gait recognition technology is instrumental in reinvigorating investigations of unsolved cases, particularly cold criminal cases that have stalled traditional methods. By analyzing unique walking patterns, it introduces a new approach that offers new leads and avenues for investigation. It allows investigators to revisit existing evidence, focusing on the movement of individuals and behaviors, potentially uncovering overlooked details or connections. Moreover, gait recognition overcomes the limitations of traditional methods by providing alternative means of identification, even in cases of obscured faces.

### Enhancement of witness testimonies (Liu et al., 2021; Lin et al., 2021)

Corroborating witness descriptions: By analyzing surveillance footage to match the gait pattern of an individual with witness-provided physical descriptions, gait recognition adds credibility to the testimonies.

Verifying movements: Gait analysis ensures alignment between witnessed movements and those observed in surveillance footage, thereby substantiating the accuracy of testimonies regarding suspect actions.

Challenging inconsistencies: Gait recognition identifies disparities between witness accounts and observed gait patterns, prompting critical examination of testimonies and potential further investigation.

Providing objective evidence: Gait analysis furnishes impartial evidence grounded in unique walking patterns, augmenting the reliability of testimonies with scientific rigor.

### Healthcare and rehabilitation (Sethi, Bharti & Prakash, 2022; Connor & Ross, 2018; Kumar et al., 2021)

Rehabilitation monitoring: To track and evaluate the progress of patients during rehabilitation programs, physical therapy settings make extensive use of gait technology. Through consistent gait analysis, therapists can objectively monitor the advancements or modifications over time. Therapists can customize treatments and therapy regimens according to the unique requirements and advancements of each patient using this data-driven approach. For example, therapists can modify exercises or therapies to address areas of weakness or imbalance, if gait analysis indicates abnormalities or uneven movement patterns. Ultimately, this customized strategy improves rehabilitation programs efficacy and aids patients in achieving the best possible recovery.

Fall detection: Defects in walking patterns can also be a sign of an elevated fall risk, especially in the senior population, where gait analysis is useful. Before a fall occurs, gait recognition technology can detect people who may be at risk of falling by examining minor changes or departures from typical gait patterns. By taking a proactive stance, healthcare professionals can reduce the likelihood of falls by implementing interventions or preventive measures, such as proposing assistive technology, making environmental modifications to eliminate dangers, or prescribing exercises to enhance balance and stability. By encouraging safety and independence, the early identification of fall risk through gait analysis of an individual improves the overall quality of life and reduces the likelihood of accidents and hospital stays.

### Sports and athletics (Sethi, Bharti & Prakash, 2022; Marsico & Mecca, 2019; Kumar et al., 2021)

Performance optimization: Athletes use gait analysis to refine their walking or running form and increase effectiveness. Gait recognition technology delves into the biomechanical elements that examine the gait pattern of an athlete under various factors such as posture, foot placement, cadence, and stride length. With these data, athletes can modify their technique to optimize energy use, minimize extraneous motions, and enhance overall efficiency. For instance, an athlete overstriding or excessive lateral movement, may be identified by gait analysis as inefficiencies in their running form. This can be corrected using focused training and technical modifications. Athletes can enhance their athletic prowess and perform better in competitions by fine-tuning their walking patterns.

Injury prevention: Coaches and sports medicine professionals use gait technology to identify biomechanical issues that may contribute to injuries, allowing for targeted interventions.

### Biomechanics research (Liu et al., 2021; Lin et al., 2021; Sethi, Bharti & Prakash, 2022; Kumar et al., 2021)

Human movement studies: Researchers use gait analysis to understand the mechanics of human movement, which is valuable for designing prosthetics, orthotics, and ergonomic products.

Clinical studies: Gait technology assists in clinical studies related to neurological disorders, musculoskeletal conditions, and other health-related research.

### Security and surveillance (Liu et al., 2021; Lin et al., 2021; Sethi, Bharti & Prakash, 2022)

Biometric identification: Gait analysis leverages distinctive walking patterns as a biometric identifier for authentication and identification. Similar to fingerprints or facial features, gait patterns are also unique and can be used as reliable markers for distinguishing one individual from another.

### Human-computer interaction (Sethi, Bharti & Prakash, 2022; Sun, Su & Fan, 2022)

Natural interaction: Integrating gait analysis into gesture recognition systems enables fluid, and instinctive interactions without traditional input devices. Users control virtual environments with subtle movements, and enhance immersion by eliminating the need for controllers.

VR immersion: Gait recognition allows users to navigate virtual worlds naturally using body movements to interact with digital content. This deepens immersion as users can walk, run, and gesture to manipulate objects and explore virtual spaces.

Gesture-based gameplay: Gait technology introduces gesture-based gameplay, translating walking patterns into in-game actions. Users can jump, crouch, or interact with objects by performing specific gestures while walking, adding physicality, and engaging in gaming.

Accessibility: Gait-based gestures enhance accessibility by offering alternative input methods to users with mobility limitations. By leveraging natural body movements, these systems are inclusive, allowing a wider range of users to participate in VR and gaming activities equally.

### Wearable technology (Sethi, Bharti & Prakash, 2022; Marsico & Mecca, 2019; Connor & Ross, 2018)

Fitness tracking: Users who wear wearable technology with gait analysis capabilities can obtain comprehensive insights into their jogging or walking habits. These gadgets provide useful information for evaluating the overall fitness and health of an individual. Users can set fitness goals, monitor their progress over time, and modify their routines, as necessary.

Assistive devices: Gait recognition technology in assistive devices like smart shoes or insoles, offers immediate feedback and aids for people with mobility impairments. These devices analyze gait patterns to deliver personalized assistance, and enhance stability, balance, and mobility. For instance, smart shoes with gait sensors can detect irregular walking patterns and provide feedback to correct the posture or stride.

### Automotive safety (Sethi, Bharti & Prakash, 2022; Marsico & Mecca, 2019)

Driver monitoring systems utilize gait analysis to evaluate driver behavior and conditions, identifying slight alterations in walking patterns that could signify fatigue or alertness. This approach enables the ongoing monitoring of driver alertness, facilitating prompt action to avert accidents. Gait analysis is particularly adept at detecting fatigue indicators, which is a notable hazard for road safety. Immediate alerts and interventions can be provided to drivers, suggesting breaks or activating automated driving modes to ensure safe vehicle operation until alertness is restored.

### Robotics (Sethi, Bharti & Prakash, 2022; Sun, Su & Fan, 2022)

Humanoid robots: Gait technology can be integrated into humanoid robot designs to facilitate lifelike walking patterns and improve the overall interaction between humans and robots. Anthropomorphic robots are machines that act more like humans. This helps people to better utilize and accept them. This also makes people feel more connected to machines, which is important for rehabilitation. Machines with human-like traits can communicate seamlessly with users. By incorporating findings from gait research, robot designers can replicate human movements, such as stride length, cadence, and posture, thereby enhancing the authenticity of the walking motion. This makes it possible for humanoid robots to engage with people in a more natural, move more adaptably through different contexts, and offer individualized physical assistance, especially in healthcare settings. Robots with gait recognition technology can predict and react to human motions by analyzing human gait patterns in real-time. This capability broadens the range of applications of robots and fosters cooperation between people and robots under a variety of circumstances.

## Different approaches for human identification using gait recognition technology

**Figure 1:**
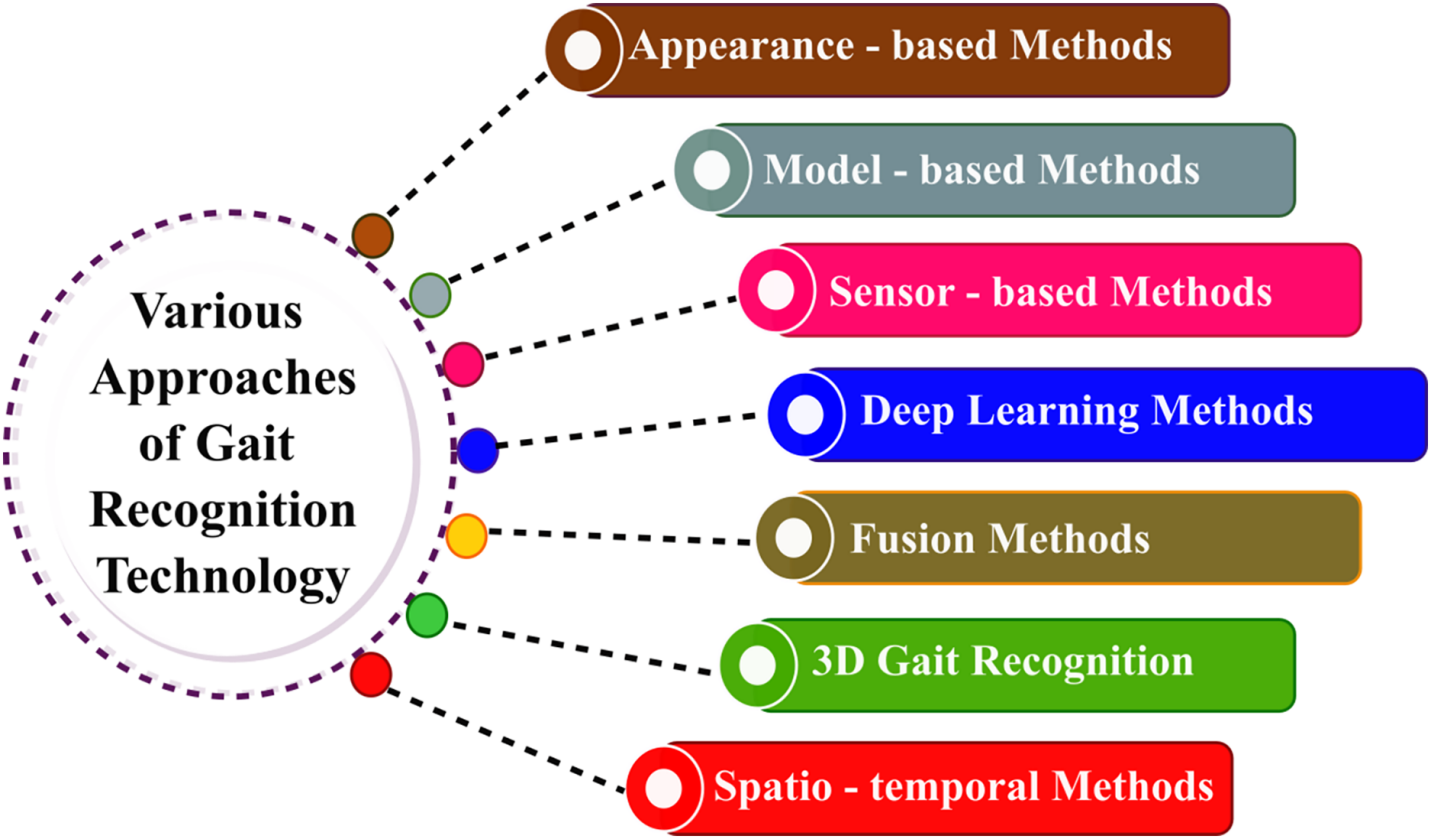
Various approaches of gait recognition technology.

Various gait recognition methodologies are addressed in this section; each methodology offers unique insights into analyzing and identifying individuals based on their walking patterns, and Fig. 1 represents the same.

### Appearance-based methods

Silhouette-based gait analysis: (Han & Bhanu, 2006; Lam, Cheung & Liu, 2011; Tao et al., 2007). This method extracts the silhouette of a person while walking and analyses the shape and motion of the silhouette. Features such as the aspect ratio, area, and height were used for recognition.

Image and video-based gait recognition: (Işık & Ekenel, 2021; Wang et al., 2010; Han et al., 2022; Mowbray & Nixon, 2003). These methods use images or videos of a person walking, and then extract features from these images or frames. This includes traditional computer vision techniques and deep learning methods.

### Model-based methods

Dynamic time warping (DTW): (Zhang et al., 2019; Bashir, Xiang & Gong, 2010). It is appropriate for gait identification because it calculates the similarity between two temporal sequences. The algorithm aligns the sequences and calculates the distance metric.

Hidden Markov models (HMMs): (Kale et al., 2004; Chen, Wu & Li, 2020; Zhang et al., 2021) HMMs model the temporal dynamics of gait patterns. Each gait sequence is represented as a sequence of states, and the model can be trained to recognize individuals based on these sequences.

Principal component analysis (PCA): (Zhang et al., 2019; Ariyanto & Nixon, 2012; Bobick & Johnson, 2001; Cunado, Nixon & Carter, 2003) PCA was used for dimensionality reduction of the gait data. It can be applied to feature extraction to reduce the complexity of the gait patterns.

### Sensor-based methods (Sethi, Bharti & Prakash 2022)

Inertial sensors: These methods use accelerometers and gyroscopes to capture motion data while walking. Machine learning algorithms can then process these data for gait recognition.

Pressure sensors: Pressure sensors embedded in the floor or insoles of shoes can capture foot pressure patterns during walking that are unique to individuals.

### Deep learning methods (Chen, Wu & Li, 2020; Zhang, Wang & Li, 2021; Ghosh, 2022)

Convolutional neural networks (CNNs): CNNs can be used for gait recognition by processing images and video data. They automatically learn relevant features from raw image data.

Recurrent neural networks (RNNs): play a role in gait identification problems because they can be used to model temporal dependencies in gait sequences.

Siamese networks: By learning to distinguish gait pattern among various people, these networks, which are made for one-shot learning tasks, can be utilized for gait recognition.

### Fusion methods (Zhang et al., 2021; Zhang et al., 2022; Cai et al., 2021; Cosma & Radoi, 2020; Teepe et al., 2021; Ghosh, 2022)

Multimodal fusion: This approach combines information from multiple sources such as video, depth sensors, and other biometric modalities (such as face or voice recognition) to improve gait recognition accuracy.

Score-level fusion: Different recognition methods, such as appearance-based and model-based, can produce scores for each individual. These scores can be fused to make a final recognition decision.

### 3D gait recognition (Sethi, Bharti & Prakash 2022)

Depth cameras: Depth cameras like Microsoft Kinect or LiDAR sensors capture three-dimensional information of a person during walking, which can be used for gait recognition.

### Spatiotemporal method (Su, Zhao & Li, 2021; Khan, Farid & Grzegorzek, 2023)

Spatiotemporal template: This method captures both spatial and temporal information regarding the movement of a person to create templates for gait recognition.

Gait energy image (GEI): GEI is a representation that combines multiple frames of silhouette into a single image, thereby emphasizing the energy distribution of the gait cycle.

- In relation to the gait recognition research in previous studies, there was a preference for model-based approaches initially (Zhang et al., 2021; Kale et al., 2004; Wagg & Nixon, 2004; Yam, Nixon & Carter, 2004; Yamauchi, Bhanu & Saito, 2010; Liao et al., 2017; Ariyanto & Nixon, 2012; Bobick & Johnson, 2001; Cunado, Nixon & Carter, 2003). However, there has been a shift towards the appearance-based methods owing to their increased popularity (Işık & Ekenel, 2021; Wang et al., 2010; Han & Bhanu, 2006; Makihara et al., 2006; Tao et al., 2007; Lam, Cheung & Liu, 2011; Wang et al., 2012; Bashir, Xiang & Gong, 2010; Mowbray & Nixon, 2003; Liu & Sarkar, 2006; Zhang et al., 2019). This shift has been driven by the ability of appearance-based methods to overcome the limitations associated with the former, such as high computational complexity, limited robustness to variations, sensitivity to environmental conditions, privacy concerns, and complex implementation.
- Appearance-based approaches offer advantages, such as reduced data requirements, lower computational complexity, robustness to variations, simplicity of implementation, and faster processing speed, making them practical for real-world applications. However, these methods have limitations, including sensitivity to environmental conditions, limited discriminative power, susceptibility to clothing variations, and privacy concerns.
- Deep learning has revolutionized gait identification by addressing these challenges through automatic feature learning, robustness to variations, transfer learning, and temporal modelling. Despite its impressive accuracy, deep learning requires a substantial amount of labelled data and can be computationally intensive, leading to ongoing research for improvement.
- Gait recognition researchers commonly employ a combination of techniques, including deep learning and spatiotemporal methods to enhance the accuracy and robustness. Sensor-based models require wearable devices, whereas appearance-based and 3D gait recognition primarily rely on visual data, with method choice influenced by the application, data accessibility, and accuracy requirements.

Researchers continue to explore and integrate various techniques to advance gait recognition. In this article, we mainly focused on the deep learning techniques and their combinations.

## Survey methodology

### Search strategy analysis

This research article draws from various databases, including SCOPUS, IEEE Xplore, Google Scholar, Web of Science, Springer, and Science Direct, covering top-indexed journals and high-impact research articles in the field from 2020 to 2023. The primary aim of the survey was to provide a comprehensive analysis of the latest developments in gait recognition for person identification, thus setting the timeframe since 2020. Relevant articles containing the keywords “gait recognition using deep learning” and “person identification” were identified without the need for additional refinement using wildcards. The focus is solely on identifying individuals based on gait recognition techniques without requiring cooperation. Consequently, the consideration of wearable devices, such as smart devices, and non-wearable devices, like sensor-based models, is excluded. Table 1 represents the keywords used to identify the research articles in their database.

**Table 1:** The keywords used in databases.

Journals	SCOPUS, IEEE xplore, google scholar, web of science, springer and science direct
Keywords	“Gait recognition using deep learning” AND “person identification”

### Inclusion and exclusion criteria

**Figure 2:**
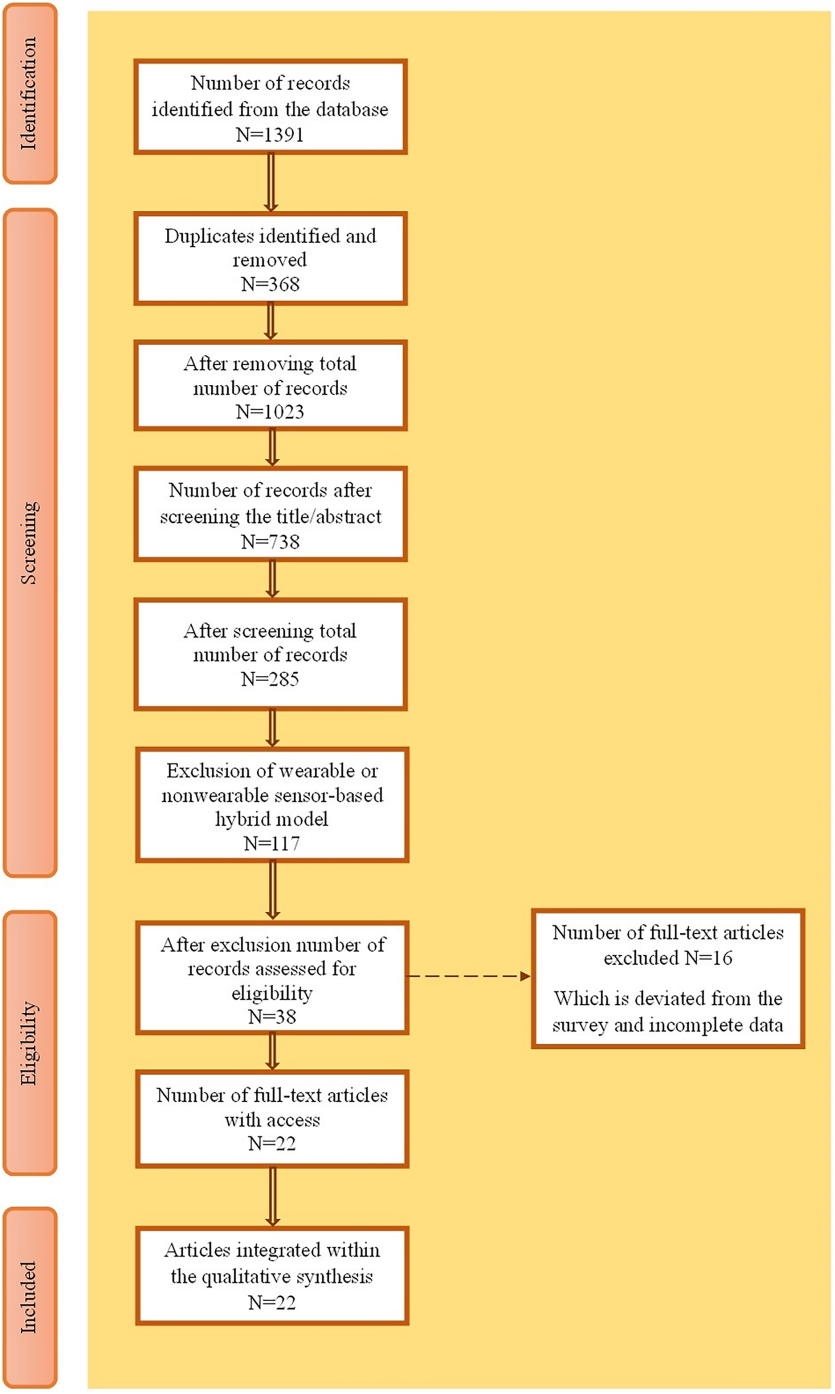
PRISMA analysis.

The inclusion and exclusion criteria while searching the research articles in their respective databases and PRISMA analysis diagrams were also framed. Figure 2 shows a PRISMA analysis diagram for the selection of articles. Table 2 shows the inclusion criteria for fine-tuning the selection process and the exclusion criteria to eliminate irrelevant topics. Of the 1,391 records, 22 were included in the qualitative synthesis after screening, eligibility assessment, and removal of duplicates and exclusions, resulting in a final selection of 22 articles.

**Table 2:** The inclusion and exclusion criteria used while searching the research article.

Inclusion criteria	Description	Exclusion criteria	Description
I1	Includes keyword as gait recognition using deep learning	E1	Exclude the wearable or nonwearable sensor-based model
I2	Studies that applied for person identification	E2	Publications which are not related to human identification
I3	Include the article between 2020 to 2023	E3	Removing the duplicates articles
I4	Include full-text articles	E4	Exclude the review and survey article
I5	Include the articles which is published in English	E5	Publication which contains incomplete data

**Note:**

Represents the inclusion and exclusion criteria while searching the research article. The criteria include recent (2020–2023) articles on gait recognition using deep learning for person identification to ensure a relevant and accurate article. Furthermore, we exclude irrelevant articles like sensor-based models, non-human identification, duplicates, reviews, and incomplete data.

### Selection of research data

**Figure 3:**
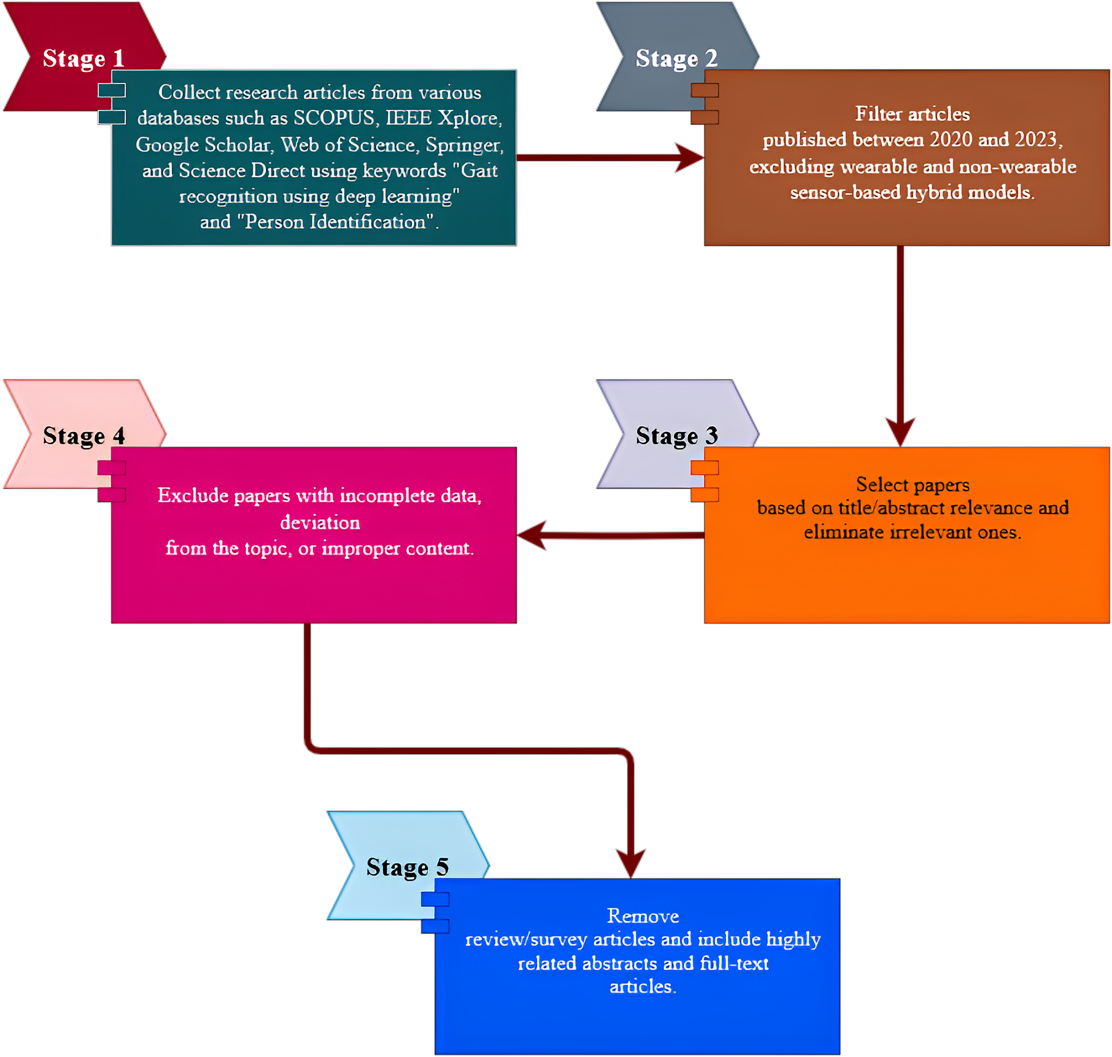
Step-by-step approaches of selection of research data.

The selection of research data can be explained using a step-by-step approach, which is also represented in Fig. 3.

Stage 1: Collect research articles from various databases such as SCOPUS, IEEE Xplore, Google Scholar, Web of Science, Springer, and Science Direct using the keywords “Gait recognition using deep learning” and “Person Identification”.

Stage 2: Filter articles published between 2020 and 2023, excluding wearable and non-wearable sensor-based hybrid models.

Stage 3: Select articles based on title/abstract relevance and eliminate irrelevant articles.

Stage 4: Articles with incomplete data, deviation from the topic, or improper content were excluded.

Stage 5: Remove reviews/survey articles and include highly related abstracts and full-text articles.

## General steps for human gait recognition system using deep learning techniques

**Figure 4:**
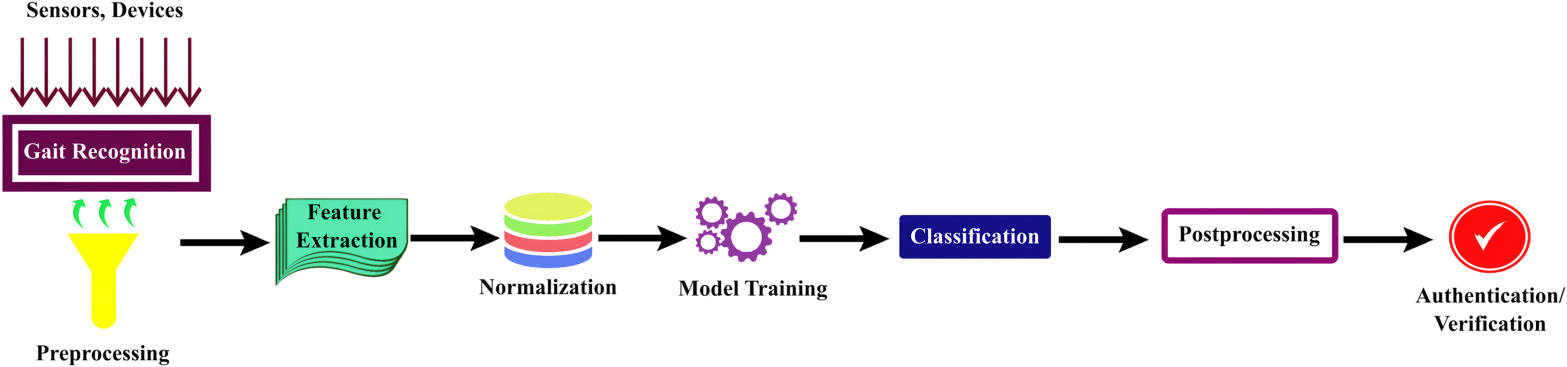
General steps for human gait recognition system using deep learning techniques.

Human gait recognition typically involves several steps. These steps can vary in complexity depending on the specific approach and technology used; however, here are the fundamental stages of human gait recognition in deep learning techniques which are shown in Fig. 4.

Gait recognition is a multi-step process that commences with

1. Data acquisition: involved the collection of video footage or sensor data to capture the walking or running patterns of an individual. Common data sources encompass surveillance cameras, depth sensors, or inertial sensors in wearable devices. Subsequently, the acquired data underwent preprocessing to enhance quality, including tasks like background subtraction and noise reduction.

2. Feature extraction: Relevant gait features are identified, such as limb motion or spatial-temporal characteristics. For feature extraction, the system may employ deep learning techniques or machine learning techniques like CNN (Chai et al., 2021; Su, Zhao & Li, 2021; Liu et al., 2021; Mu et al., 2020; Nixon, Tan & Chellappa, 2010), RNN (Ghosh, 2022), Mask R-CNN (Zhang et al., 2021, 2022), ResNet (Cosma & Radoi, 2020), long short-term memory (LSTM) (Cosma & Radoi, 2020; Zhang et al., 2022), and other techniques like the Baum-Welch Algorithm or GEI (Chai et al., 2021; Inui et al., 2020), which aid in extracting discriminative features.

3. Normalization: The discriminative features are then normalized using techniques like batch normalization to account for variations in speed and size.

4. Model training: The core of the process is model training, during which machine learning or deep learning models learn to recognize unique gait patterns from a labeled dataset.

5. Classification: Once trained, these models were employed for the recognition or classification of new gait data. Various loss functions, such as reconstruction loss (Chai et al., 2021; Zhang et al., 2022), triplet loss (Zhang et al., 2022; Su, Zhao & Li, 2021; Schroff, Kalenichenko & Philbin, 2015; Fan et al., 2020; Chao et al., 2019; Hermans, Beyer & Leibe, 2017), identity similarity loss (Chai et al., 2021), cross-entropy loss (Li et al., 2020), softmax function (Ghosh, 2022), cosine similarity loss (Chaudhari, Pawar & Deore, 2013; Connor & Ross, 2018; Hinton, Vinyals & Dean, 2014), and angular loss functions (Kumar et al., 2021; Marsico & Mecca, 2019) are used for classification, either individually or in combination.

6. Post-processing: This step refines the results, and gait patterns are frequently used for authentication or verification in real-world applications. The system performance is evaluated, and continuous learning may be implemented to adapt to changing gait patterns over time, rendering gait recognition a dynamic and evolving field in biometrics and security.

## Literature review on human activity recognition

In this section, we discuss, various deep learning models such as CNN, Mask R-CNN, CNN-LSTM, LSTM-ResNet, GCN, CNN-LSTM-GCN hybrid, silhouette-skeleton-based gait representation, fully connected network, hidden Markov model, GI-ReID, and Faster R-CNN-RNN combination. Additionally, a comparison table (Table 3) is provided to illustrate various gait recognition approaches.

**Table 3:** Comparative analysis of various gait recognition approaches.

Methods used	Limitations	Advantages	Dataset used	Loss function	Reference
(1.1) Gate controlled and shared attention ICDNet (GA-ICDNet)	It may not be robust to illumination and background changes.	Proposed modules improve recognition accuracy in gait recognition. Shared attention module and covariate feature control gate module address over-disentanglement. The model provides a more fine-grained representation at spatial and channel aspects.	OU-LP Bag, OU-Lp Bag-β	Reconstruction loss, triplet loss, identity similarity loss and cross entropy error loss.	Chai et al. (2021)
(1.2) Spatio-temporal representation model	It might not be resilient to alterations in clothing and perspectives, potentially impacting how gait appears and moves.	Refines spatial features to extract more discriminative features Deals with variations caused by walking speed, carrying, dressing condition, and viewpoint Provides solutions for gait recognition in real-world scenarios	CASIA-B	Triplet loss function	Su, Zhao & Li (2021)
	It does not consider the effects of clothing variations, occlusions, and noise on gait recognition, which may reduce its robustness and generalization.	SelfGait utilizes massive, diverse, unlabeled gait data for pre-training. It improves the representation abilities of spatiotemporal backbones. It is effective for subjects with coat or jacket.	CASIA-B OU-MVLP	Cousine similarity Loss	Liu et al. (2021)
(1.3) iLGaCo model	It relies on a limited set of past samples to preserve prior knowledge, which might be lacking or inadequate in certain situations, and only accounts for two covariate factors: viewpoints and walking conditions.	iLGaCo allows for incremental learning of covariate factors for gait recognition. It addresses catastrophic forgetting by using a small memory for previous samples. The approach achieves competitive performance and can be applied to add new information. It has limited storage requirements and low computational cost.	CASIA-B	Cross-entropy loss and hinton loss	Mu et al. (2020)
(1.4) Multi-temporal-scale feature extractor (MSTFE) model	It overlooks the spatial details of gait, like global and local body features, potentially compromising its resilience to variations in clothing, carrying conditions, or viewing angles.	Extracts gait features from multiple temporal scales Integrates slowly changing and rapidly changing gait information Improves performance in complex carrying conditions	CASIA-B, outdoor gait	Triplet loss function and cross entropy loss	Lin et al. (2021)
(1.5) Cross-view gait recognition model	It may not perform well on low-quality silhouettes.	Global features focus on the subject's shape, while local features extract fine-grained information.	CASIA-B	Triplet loss function	Hong et al. (2021)
(1.6) Deep convolutional neural network model	It may not cover all the possible variations and challenges in gait recognition, such as different clothing, lighting, and background conditions.	Enables recognition from long distance and low-resolution images Fine-tuning of pre-trained model improves gait recognition performance	CASIA-B, OU-ISIR	–	Işık & Ekenel (2021)
	The proposed CNN model is only evaluated on the CASIA-B gait dataset, which may not be representative of real-world scenarios or other gait dataset and does not consider the effects of other factors that may affect gait recognition, such as occlusion.	Gait Energy Images (GEI) capture spatiotemporal gait information for identification. Proposed method shows effectiveness in environments with clothes changes and viewing angles variation.	CASIA-B	Cross entropy loss and softmax loss	Aung & Pluempitiwiriyawej (2020)
(1.7) Global and local feature extraction model	It does not address the challenges of cross-view, noise, occlusion, or low resolution in real-world scenarios.	Utilizes both global visual information and local region details. Introduces Local Temporal Aggregation to preserve spatial information. Improves discriminativeness of visual features for better gait recognition performance.	CASIA-B, OU-MVLP	Triplet loss function and cross entropy loss	Lin, Zhang & Yu (2021)
(1.8) Combination of mask R-CNN and CNN model	It might not be resistant to variations in lighting and background conditions.	Gait recognition based on gait features can be collected under long-distance and contactless conditions.	CASIA-B	Triplet loss function and softmax function	Zhang et al. (2021)
	The number of subjects is small (only four people) and the gait data is not collected in a specified place or condition, which may affect the generalization and robustness of the proposed method. It only tested on two patterns of gait (with nothing and with a bag).	It improves the accuracy and robustness of gait recognition in real environments, where the background and the foreground are often complex and dynamic. It can also reduce the computational cost and storage requirement of gait recognition, as only one GEI image is needed for each person.	CASIA-B	–	Inui et al. (2020)
(1.9) Combination of mask R-CNN, CNN and LSTM model	It might struggle with extreme changes like occlusion, lighting, and pose, and might not adequately capture the dynamic aspects of gait crucial for distinguishing individuals.	GaitNet disentangles appearance, canonical, and pose features for better recognition. Gait biometrics identification has advantages in long-distance and lower resolution scenarios.	Front-view gait (FVG)	Cross reconstruction loss, pose similarity loss, canonical similarity loss, incremental identity loss	Zhang et al. (2022)
(1.10) Combination of LSTM and ResNet model	It may not be robust to clothing changes and view changes, which can affect the appearance and motion of gait.	MFINet method incorporates confounding factors in the decision process. MFINet achieves an accuracy of over 85% in scenarios with the same angle. MFINet uses a skeleton image representation to capture temporal dynamics.	CASIA-B	Triplet loss function and cross entropy loss	Cosma & Radoi (2020)
(2.1) GCN model	Recognizing gait in UAVs is challenging due to pitch rotations, and data quality can be affected by factors like clothing changes and carrying objects.	Gait recognition using UAVs provides extended operational ranges and adaptability, requiring minimal data quality and no cooperation.	UAV-gait	–	Ding et al. (2022)
(2.2) Combination of ResGCN and CNN model	It does not analyze the robustness or generalization of the proposed method to different pose estimation errors, occlusions, or variations in gait speed, stride length, or style. The article does not provide any qualitative or visual results to illustrate the learned gait features or the effect of the spatial and temporal modeling.	Skeleton poses from RGB images offer robustness and accuracy, particularly in complex scenes. Representing poses as a graph captures spatial and temporal relations between joints and limbs. GCNs handle variable-length sequences and incorporate global and local information effectively.	CASIA-B	–	Teepe et al. (2021)
(2.3) Combination of CNN, LSTM and GCN model	Effectiveness hinges on pose estimation accuracy, which may falter in certain situations, particularly when capturing overlapped body structures during walking, necessitating intricate model and fusion approaches for optimal integration of silhouette and skeleton data.	Heatmap for joints achieves better performance than other skeleton representations. Proposed approach boosts performance of both set-based and sequence-based gait recognition.	CASIA-B	Triplet loss function and cross entropy loss	Cai et al. (2021)
(3) Fully connected network model	It overlooks the changes in gait appearance caused by clothing, carrying conditions, or different speeds, potentially impacting recognition accuracy.	Solid physical interpretability for gait recognition system. Effective method for cross-view gait recognition on large dataset.	OU-MVLP	Autoencoder, triplet loss and softmax loss function	Zhang et al. (2021)
	The method requires a large amount of training data to learn the angular softmax loss and the triplet loss, which may not be available in some scenarios. The method does not consider the temporal dynamics of gait, which may contain useful information for cross-view recognition.	A-Softmax loss helps in learning a separable feature representation in the angular space. BA triple loss makes the learned feature representations more discriminative.	CASIA-B, TUM GAID database	Angular loss and triplet loss function	Han et al. (2022)
(4) Temporal part-based GaitPart model	It may not perform well on low-quality silhouettes.	GaitPart model enhances fine-grained learning of spatial features. Micro-motion Capture Module (MCM) focuses on short-range temporal features. GaitPart achieves state-of-the-art performance on multiple standard benchmarks.	CASIA-B, OU-MVLP	Triplet loss function	Fan et al. (2020)
(5) Hidden markov model	It does not consider the appearance variations of gait due to clothing, carrying condition, or varying pace, which may affect the recognition performance.	Gait recognition based on GFHI provides a compact representation of human motion. Increasing the number of views in the training data improves recognition accuracy.	USF, CMU MoBo, CASIA, self-built gait datasets	–	Chen, Wu & Li (2020)
(6) GI-ReID model	Imperfect gait predictions due to occlusion and multi-person scenarios. Challenges in capturing perfect gait results due to environmental differences.	Gait-stream captures cloth-invariant biometric characteristics, reduces computing costs, and excels in cloth-independent gait recognition	Real28, VC-Clothes, LTCC, PRCC	Classification loss and triplet loss	Jin et al. (2022)
(7) Combination of faster R-CNN and RNN	Gait recognition systems struggle with various walking patterns due to COs. Challenges include object characteristics, moving objects, and view angles.	Faster R-CNN outperforms YOLOv3 and Cascade R-CNN in pedestrian detection. Bayesian optimization technique used for optimal hyperparameter selection. Novel method for gait recognition with and without carried objects.	OU-LP-Bag OUTD-B OULP-Age CASIA-B	Classification loss and regression loss	Ghosh (2022)

### CNN based models

A CNN is a deep learning model that specializes in handling visual data, such as images and videos, with exceptional effectiveness. This section provides an overview of several models based on CNNs that are used for human gait recognition, which is the process of recognizing people based on the way they walk. The distinct methodologies of each subsection are explained, including the disentangled framework of GA-ICDNet (Chai et al., 2021), the incremental learning technique of iLGaCo (Mu et al., 2020), and the many ways in which CNN, LSTM, and Mask R-CNN are combined to process and extract features. These models enhance the gait identification performance on a variety of datasets and in practical applications by employing distinct loss functions and feature extraction methods.

#### GA-ICDNet based model

Gate Controlled and Shared Attention ICDNet (GA-ICDNet) was introduced in the work published by Chai et al. (2021). The proposed system was based on the disentangled framework representation proposed in the work published by Li et al. (2020). GA-ICDNet has an encoder that differentiates the image into identity and covariate parts, a shared attention model-introduced semantic label that helps identify the covariate position at the spatial aspects, a control gate generation model that provides detailed information of the covariate, and a decoder that recovers the GEIs with and without covariates. The study proposed by Chai et al. (2021), used four types of loss functions to compute the distance between the current output and the expected output of the algorithm, including reconstruction loss, triplet loss, identity similarity loss, and cross-entropy error loss.

#### Spatio-temporal representation model

Su, Zhao & Li (2021) proposed, multiple spatiotemporal feature extraction, temporal pooling, and horizontal pooling. Three modules make up each spatiotemporal feature extraction: The Micro Information Integration (MII) module additionally incorporates temporal information and amplifies the receptive field in the time dimension. A CNN extracts features frame-by-frame. Temporal Information Passing (TIP) assists in identifying the temporal and spatial features between different frames extracted from the CNN. The average gait image period, or temporal pooling, represents both the spatial and temporal information. The feature map is uniformly divided into horizontal stripes by a horizontal pooling module, which also normalizes each column vector inside a stripe to a single column vector to provide fine-grained information. Su, Zhao & Li (2021) exclusively employed the triplet loss function.

The pretraining pipeline of SelfGait (Liu et al., 2021) adopts two major networks: an online network and a target network, which act as a backbone for extracting features in spatiotemporal techniques. An online network consists of an online encoder, transition model, online projection, and prediction. The online encoder has Horizontal Pyramid Mapping (HPM), which converts all feature frames into patches for tracing multi-scale spatial features. Then, it undergoes a transition model that acquires knowledge about temporal features. Online projection and prediction were allocated to the identity feature, and output of the online network. The pipeline of the target network has a target encoder and target projection, which are similar to the online encoder and online projection.

#### iLGaCo model

Mu et al. (2020) developed iLGaCo, which combines a method to prevent catastrophic forgetting with an incremental learning strategy for covariates used in gait identification. The algorithm uses CNN and the GaitSet model (Chao et al., 2019) as an incremental learning end-to-end approach. The model undergoes a streamlined training procedure using fresh data and a limited fraction of samples from the past. The training procedure, as well as the selection and memory updates, comprised the two primary components of iLGaCo. First, samples from memory and fresh samples were combined to train a CNN model. In the second stage, the samples stored in memory are updated by the selection algorithm. It has two loss functions in its architecture, the Hinton, Vinyals & Dean (2014), integrated for network compression, and cross-entropy loss for classification.

#### Multitemporal-scale feature extractor model

The proposed method for gait recognition uses a spatial-temporal feature extraction process, incorporating a multi-scale temporal feature extractor (MSTFE) (Lin et al., 2021), and subsequent operations for temporal aggregation and spatial mapping. The model extracts spatial features from the inputs using 2D and 3D convolutions and aggregates temporal information using a multi-scale temporal feature extractor (MSTFE). The method then performs temporal aggregation (Lin, Zhang & Bao, 2020; Lin et al., 2020) and feature mapping, integrating temporal information across the entire sequence and splitting feature maps into horizontal strips (Fan et al., 2020; Chao et al., 2019). The training stage involves a combined loss function for optimization, whereas the test stage evaluates accuracy using a gallery-probe matching approach. This method effectively recognizes gait patterns in video sequences.

#### Cross-view recognition model

Hong et al. (2021) introduced cross-view gait recognition based on feature fusion. Multiscale fusion extracts the features from different receptive fields. Subsequently, it undergoes a dual-path structure. The global feature module identifies the characteristics of color, texture, and shape features; here, it is mainly concentrated on shape features. In the case of aliasing and flawed pictures, local features are more stable. The proposed method utilizes an attention-based Temporal Feature Aggregator to capture both the intricate local micro-motion features and the overarching description of the complete gait sequence, aiming to enhance temporal feature extraction (Fan et al., 2020). To assess the training progress, the triplet loss function (Schroff, Kalenichenko & Philbin, 2015) was introduced, which ensures that instances from the same class are positioned closer to each other in the feature space than instances from different classes.

#### Deep convolutional neural network model

The approach outlined by Işık & Ekenel (2021) encompassed multiple stages. During the preprocessing stage, the positions of the silhouettes in the frames were selected. It uses cropped and centered silhouettes. The aspect ratio of the cropped silhouettes was preserved by zero-paging all photos to 128 × 88 size. The deep convolutional neural network can handle RGB full-body human images and binary human silhouettes. The first phase uses several convolution layers, and then pooling layers are used to remove the characteristics of the image. The feature representation from the first phase, which is fed into the next phase of the network, acts as the classifier. Updating the fully linked layers using the new classification aim and pre-trained feature extraction component in its existing configuration are alternatives for transfer learning. The feature vectors for a gait sequence were extracted from each frame, and element-wise max pooling was performed on each feature vector. The cosine similarity measure was used by the algorithm to assess the degree of similarity between the gallery and probing feature vectors.

CNNs (Nixon, Tan & Chellappa, 2010) are among the most widely used and prominent deep learning techniques. It is mostly used in computer vision techniques, such as object detection, image processing, and video processing. It has the fewest possible layers and parameters as well as a very short training period. In the model proposed by Aung & Pluempitiwiriyawej (2020), deep convolutional neural networks were employed to determine the essential gait characteristics for human identification. Both the cross-entropy and softmax loss functions were used in the network. The error between the predicted classification and the actual value was measured using cross-entropy loss as the loss function in softmax.

#### Global and local feature extraction model

In the work published by Lin, Zhang & Yu (2021) exploited, the integration of global-local feature representation (GLFA) and local temporal aggregation (LTA). LTA can preserve spatial details by reducing temporal precision to achieve a heightened spatial level of detail. The GLConv layer, which includes both global and local feature extractors, allows implementation of the GLFE module. The local feature extractor is used to extract more information from localized feature maps, whereas the global feature extractor can extract all gait-related data. Owing to various combinations, GLConv has two distinct structures: GLConvA and GLConvB. “GLConvA-SP-GLConvA-GLConvB” are the four layers that make up the GLFE module. In this study, generalized-mean pooling was used to incorporate the spatial information. Cross-entropy loss can be used to identify Human IDs using triplet loss (Fan et al., 2020; Chao et al., 2019; Hermans, Beyer & Leibe, 2017) by increasing the inter-class distance and decreasing the intra-class distance.

#### Combination of mask R-CNN and CNN model

V-HPM is a combination of horizontal pyramid mapping (HPM) and vertical pyramid pooling (VPP) and was proposed in a previous study by Zhang et al. (2021) for feature extraction. Initially, Mask R-CNN extracts the human gait silhouettes and has a feature extraction network, region proposal network (RPN), region of interest (RoI) align part, and segmentation part, which can be carried out using a two-stage detection and segmentation model. Subsequently, an improved Gaitset algorithm was introduced with V-HPM, which helps identify the relationship between the image sequences by itself without any time frames. The softmax and triplet loss functions were used for joint training of the loss function.

A CNN is composed of a convolutional layer, pooling layer, normalization layer, two successive triples, and two fully connected layers. Inui et al. (2020) combined GEI and CNN approaches for gait recognition. The GEI approach was used to obtain significant results. The GEI of CASIA Dataset B was trained and classified using a deep CNN constructed by the researchers. Batch normalization is used to address network purchase loss, speed up learning, simplify parameter adjustment, and stabilize the distribution of each layer of data in the network. It also helps to enhance the optimization efficiency. The suggested model consists of eight feature maps, four convolutional layers, four pooling layers, and batch normalization applied after each convolutional layer.

#### Combination of mask R-CNN, CNN, and LSTM model

The output of the disentanglement was proposed by Zhang et al. (2022) and made possible by GaitNet. GaitNet is an autoencoder implemented based on a CNN with distinctive loss functions. The encoder estimates three latent representations for each video frame: posture, which highlights the placement of the body parts, canonical, which describes the interesting characteristics of each particular body component; and appearance features, which depict the attire of the subject. (1) Cross reconstruction loss implies that the final option outline may be decoded from the accepted additionally, appearance highlights of one casing combined with the posture element of another edge; (2) Canonical consistency loss favors movies of the same subject taken under varied conditions that have the same canonical qualities; and (3) Pose similarity loss causes a succession of pose features derived from a video of the same subject to appear identically regardless of the situation. The sequence-based dynamic gait feature was created by feeding the pose characteristics from the sequence into a multi-layer LSTM with the expected gradual identity loss.

#### Combination of LSTM and ResNet model

The method for gait recognition proposed by Cosma & Radoi (2020) was MFINet (Multiple Factor Inference Network) which is based on skeletal sequences derived using posture estimation methods (Insafutdinov et al., 2016). Confounding elements (such as angle and movement fluctuations) are learned by MFINet while performing other tasks, which adds more details to the recognition process. It has several phases. Extracting 2D skeletons from monocular photos is the initial step. This was performed using DeeperCut, which is a well-known open-source framework created by Insafutdinov et al. (2016). The model, which was trained using Microsoft COCO (Lin et al., 2014), performed well on benchmarks for 2D posture estimation, and was represented by a list of skeletons in chronological order. The Stacked Dilated Convolution Blocks in the feature extractor network, which represent skeletons and maintain both the temporal and spatial structure of human walking, traverse the TSSI images. Concatenating the generated feature maps yields a single block. Residual connections are used to speed up the gradient propagation and incorporate more low-level characteristics. This method of creating skeletons safeguards the temporal and spatial structures of a person walking.

### GCN based models

Neural networks with graph convolutional networks (GCNs) have been specifically engineered to handle graph-structured input. In gait recognition, GCNs play a key role in analyzing walking patterns expressed as graphs, where nodes correspond to component features (*e.g*., joint positions) and edges denote the relationships between these features. By aggregating information from neighboring nodes and edges, GCNs enable the extraction of meaningful gait features that capture both the spatial and temporal characteristics of walking patterns. In this section, research has explored gait recognition from UAVs at altitudes of 10, 20, and 30 m using innovative models: one integrates graph convolution with traditional CNNs, another combines skeleton poses with a GCN, and a third merges CNN, LSTM, and GCN for hybrid silhouette-skeleton gait representation. These approaches enhance feature extraction and spatiotemporal representation, and improve gait recognition accuracy.

#### GCN model

In general, research has focused on identifying gaits using only data from cameras positioned from 1 to 5 m above the ground. The initial approach of this research aims to address the absence of gait recognition data for Unmanned Aerial Vehicles (UAVs) by compiling ground-level and UAV-based gait data to create a UAV-Gait dataset. This dataset includes data captured at altitudes of 10, 20, and 30 m during flight. The system proposed by Ding et al. (2022) consists of two pipelines, the main pipeline (MP) and multilayer global pipeline (MGP), to extract gait features. While MGP uses Set Pooling (SP) to extract global characteristics from the entire sequence, MP uses 2D convolution to obtain spatial data from each frame. To better capture the local form detail and alleviate feature misalignment, innovate by using graph convolution-based part feature pooling. Subsequently, a discriminative space is created from these characteristics using completely connected layers and Horizontal Pyramid Mapping (HPP). Using graph convolution, they aggregated neighboring part features either spatially or temporally in their proposed graph convolution-based part feature pooling. It builds a graph in which nodes represent component features, and edges are defined by how similar features are to one another. The graph was then subjected to graph convolution, and max pooling was used to extract the final component features.

#### Combination of ResGCN and CNN model

A novel technique called GaitGraph was introduced in the work published by Teepe et al. (2021). GaitGraph is a modern model-based approach, used to recognize gait features. This method seamlessly combines skeleton poses with GCN. GaitGraph uses the original image and employs a pose estimator to calculate the human pose in each frame. It utilizes HRNet (Sun et al., 2019) to accurately estimate and localize a 2D human pose, relying on the state-of-the-art approach proposed by Cheng et al. (2020) to create a heatmap with 17 key points. The ResGCN (Song et al., 2020) block is a composite structure of a residual connection with a potential bottleneck structure, followed by a traditional 2D temporal convolution and a graph convolution to enhance the extraction of refined gait information.

#### Combination of CNN, LSTM, and GCN model

A hybrid silhouette-skeleton-based gait representation was implemented by Cai et al. (2021). The input of the RGB-colored image extracts two features, the silhouette and skeleton heatmap for each frame based on pose estimation. These two extracted features were integrated into a single silhouette-skeleton image during the fusion process for gait recognition. The single compact silhouette-skeleton image was then subjected to a Gait embedding network, which adopts the GaitPart (Fan et al., 2020) and consists of two main components: Frame-level Part Feature Extraction (FPFE) and Micro-motion Capture Module (MCM). The extraction of the part-educated spatial elements for each frame is the responsibility of the FPFE. The MCM, which is also known as the Micro-motion Capture Module, as its name suggests, focuses on spatiotemporal representations of the related portion. The system uses a combination of triplet and entropy losses as the loss function.

### Fully connected network model

Zhang et al. (2021) introduced a model which is composed of three primary components: the initial component is an Observation Function Approximating module or OFA, followed by a Koopman Matrix Memory or KMM, and finally, the system includes a Discriminative Feature Extractor module or DFE. The OFA (Kingma & Welling, 2013) fed the input image to an encoder, and the encoder used a non-linear deep network transformation to convert the original input data into Koopman space. To prevent the code in the Koopman space from converging on outliers, such as zeros, and to maintain the majority of useful information in the original images, a decoder is used. Koopman Matrix Memory (KMM) (Rowley et al., 2009; Williams, Kevrekidis & Rowley, 2015) can be used for system state analysis by expressing a nonlinear dynamical system in a linear space using the Koopman operator. Finally, the committed Koopman matrix was transformed into a fresh feature within a discriminative space using a simple fully connected network. The DFE module uses softmax loss and triplet loss with hard mining (Hermans, Beyer & Leibe, 2017) as its two identity identification methods.

GaitSet employed by Han et al. (2022), is composed of a combination of different losses and has two stages: the training stage and the testing stage. Alignment and network training are the two key stages of the training process. The testing procedure consisted of three steps: feature extraction, feature alignment, and gallery search. Similar to the training stage, the silhouettes were first set to a predetermined size. The trained network was then probed with walking silhouettes to extract gait features. For verification, the closest neighbor was measured against a predefined threshold. The identification of the nearest neighbor is the outcome of the recognition if the distance is less than the threshold. A-Softmax loss (Liu et al., 2017) and triplet loss were used to train the suggested model simultaneously. While the A-Softmax loss applies a decrease in intra-class distance and an increase in inter-class distance to capture discriminative traits, the triplet loss of Hermans, Beyer & Leibe (2017) imposes an angular margin to extract separable features. A batch-normalization layer (Luo et al., 2019) was used to enable the training process once the features were extracted.

### Temporal part-based GaitPart model

In the work published by Fan et al. (2020), the integration of FPFE, which stands for Frame-level Part Feature Extractor, and TFA, which stands for the Temporal Feature Aggregator was carried out. The FPFE extracts spatial features that consider specific body parts for each frame. This is achieved through a convolutional network comprising three blocks, where each block comprises a pair of FConv or Focal Convolution Layer. The feature map undergoes horizontal division into n segments using the Horizontal Pooling (HP) module, which is designed to capture distinctive part-specific features of human body segments. The work of modeling the short-range spatiotemporal properties of each corresponding body segment was divided across n parallel MCMs within the TFA. The loss function employed here is a Separate TripletLoss.

### Hidden markov model

The HMM is an easy-to-understand model that can effectively categorize data and appropriately describe a dynamic time series. In the work published by Chen, Wu & Li (2020), a combined HMM and GFHI or Hidden Markov Model and Gait Optical Flow History Image were used. The Motion History Image (MHI) and the optical flow grayscale image serve as the foundation for the GFHI (Horn & Schunck, 1980). The optical flow can be used to calculate the local movement speed of the target. Because the MHI is a grayscale image containing temporal information, the pixel value for the most recent motion is higher. The network uses Hu Invariant Moment Feature Extraction (Hu, 1962) to identify the local details in the image.

### GI-ReID model

The main goal of Jin et al. (2022) was to recognize the same individual repeatedly in different contexts. CC-ReID represents cloth-changing person re-identification whereas the GI-ReID system makes every effort to fully utilize the characteristic human gait to resolve the cloth-changing challenge of ReID by using solely one image. The GI-ReID system utilizes a dual-stream framework that consists of an image ReID-Stream and an extra stream designed for gait recognition, referred to as Gait-Stream. These two streams, ReID-Stream and Gait-Stream, were trained simultaneously while adhering to a high-level semantic consistency (SC) constraint. The learning of cloth-independent feature sets is regulated by the gait characteristics in the ReID-Stream. The gait stream consisted of two components: a pre-trained gait recognition network, GaitSet (Chao et al., 2019), and a gait sequence prediction module. The purpose of the GSP or gait sequence prediction module is to enhance the gait data. The training of ReID-Stream is then guided by GaitSet using augmented gait features that are both independent of clothing and discriminative in terms of motion signals.

### Combination of faster R-CNN and RNN

Implementing a modified Faster R-CNN architecture, the proposed method by Ghosh (2022) first determines whether a human is present in any of the frames of the walking activity video. Instead of the five convolutional layers of the classic Faster R-CNNs, nine distinct convolutional layers were used in this enhanced version. The aspect ratios of the anchor boxes were modified to accommodate walkers with various widths and heights. The RPN uses a tiny network to create region suggestions. The feature matrix resulting from the final convolutional layer is slid over by using a single window. A 256-D network was mapped to each sliding window to acquire spatial information for each site. The number of region proposals projected for each location depended on the number of anchors in a single sliding window. Nine anchor boxes were used for each site in this work, which employing three scales and three aspect ratios. The RPN creates region proposals with five-tuple values (index, x, y, w, h) of different sizes. The RoI or Region of Interest pooling layer projects the feature matrix generated from the final convolutional layer onto it. Among the positive suggestions labeled by the RPN, this layer selects 100 RoI proposals that exhibit higher Intersection over Union (IoU) values for this specific inquiry. Furthermore, the RoI pooling layer employs max-pooling on the feature matrix corresponding to each proposal. The proposed architecture simultaneously accomplishes two tasks: it assesses whether a pedestrian is present in the frame and it establishes a bounding box around the detected individual. Every region of interest (RoI) obtained from the RoI pooling layer undergoes individual processing by the fully connected (FC) layers. The output layer employs the softmax function to forecast the existence of a human, and a regressor to generate coordinates for a rectangular box enclosing the identified individual. The detection network calculated the classification and regression losses in the output layer. These losses must be calculated to identify the presence of a person and establish a bounding box.

## Datasets

**Figure 5:**
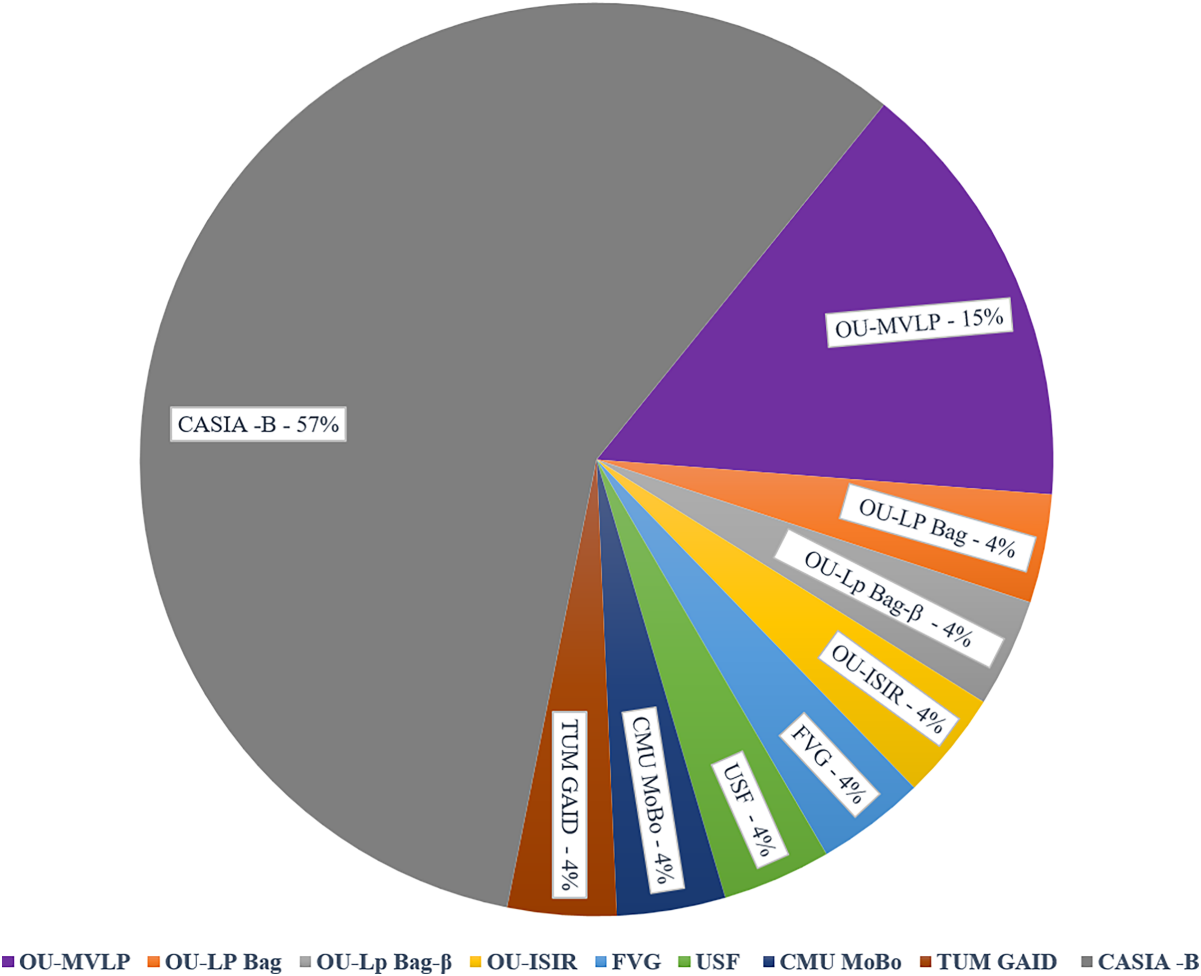
Percentage distribution of gait recognition datasets across different studies.

This section offers a comprehensive overview of the various gait recognition datasets used in the research, including details such as dataset sizes, sources, and characteristics. These datasets vary in terms of subjects, recording conditions, and privacy considerations, providing diverse resources for gait analysis studies. Table 4 explains the number of subjects utilized in GAIT datasets, along with their corresponding counts. Figure 5 illustrates the Percentage Distribution of Gait Recognition Datasets across Studies and Publications. The CASIA-B dataset was the most commonly used, accounting for 57% of the total dataset. It includes the gait sequences of 124 subjects from 11 different angles and meets the needs of most models. The OU-MVLP dataset had a 15% usage rate. Other datasets, including the OU-LP Bag, OU-LP Bag-β, OU-ISIR, FVG, USF, CMU MoBo, and TUM GAID, each constituted 4% of the usage. These less frequently used datasets can be selected based on the specific system requirements.

**Table 4:** Commonly employed subjects in GAIT datasets and their numbers.

No.	Datasets name	No. of subjects	Reference
1	OU-MVLP	10,307	Takemura et al. (2018)
2	OU-LP Bag	62,528	Makihara et al. (2017)
3	OU-Lp Bag-β	2,070	Makihara et al. (2017)
4	OU-ISIR	1. Treadmill: 168	Makihara et al. (2012)
		2. Large population: 4,007	
5	Front-View Gait (FVG)	226	Zhang et al. (2019)
6	USF	122	Sarkar et al. (2005)
7	CMU MoBo	25	Gross & Shi (2001)
8	TUM GAID database	305	Hofmann et al. (2014)
9	CASIA	124	Zheng et al. (2011)

### OU-MVLP: Takemura et al. (2018)

Data source: A science museum protracted video-based gait analysis presentation yielded the data. All participants willingly consented to participate in the study.

Dataset size and diversity: The dataset encompasses 10,307 individuals, including 5,114 males and 5,193 females, covering a broad age spectrum from 2 to 87 years.

Multiple viewing angles: Gait images were captured from 14 distinct viewing angles, spanning from 0° to 90° and 180° to 270°, providing comprehensive coverage for gait analysis.

Image quality and frame rate: Image resolution: 1,280 × 980 pixels and Frame rate: 25 frames/seconds.

Camera configuration: Seven network cameras were strategically placed at 15-degree intervals along a quarter-circle path, with their centers coinciding with the walking area. This path had a radius of approximately 8 m and a height of approximately 5 m, enabling varied gait perspectives of the subjects.

### OU-LP-Bag: Makihara et al. (2017)

This dataset was generated during a hands-on exhibition on video-based gait analysis at the Miraikan Science Museum, where visitors electronically provided consent for research. It included 62,528 subjects with ages spanning from 2 to 95 years. The camera setup ensured clear and detailed images, which allowed for comprehensive gait analysis with an image resolution of 1,280 × 980 pixels and a frame rate of 25 frames/second.

###  OU-LP-Bag β: Makihara et al. (2017)

Training set: This includes 2,068 sequences representing 1,034 individuals, with each person having two sequences, one with and one without carried objects.

Probe set: Comprising 1,036 subjects, this set features individuals who carry objects that are distinct from the training set.

Gallery set: The gallery set mirrors the probe set subjects, but shows them without carrying objects.

### Front-view gait: Zhang et al. (2019)

Database creation: This database was assembled during the years 2017 and 2018 to support research in the field of gait recognition.

Privacy enhancement: To protect subject privacy, a modified version called front-view gait (FGV)-B was created, which blur facial features to a level where even advanced face recognition algorithms fail to identify individuals.

Challenging frontal view: Frontal-view walking is considered more challenging in gait recognition because it provides limited gait cues compared with other view angles.

Diverse conditions: FVG-B encompasses diverse conditions, including variations in walking speed, carried items, and clothing, all captured from a frontal perspective.

Database details: FVG-B included frontal walking videos from 226 subjects, with 12 subjects being recorded in both 2017 and 2018, resulting in a total of 2,856 videos. A tripod at a height of 1.5 m was used to hold either a GoPro Hero 5 or Logitech C920 Pro webcam to record the movies. They had a resolution of 1,080 × 1,920 pixels and an average duration of 10 s.

### USF: Sarkar et al. (2005)

The USF Human ID Gait Challenge Dataset is a collection of video data designed for gait recognition research. It contains videos of 122 subjects, and these subjects can be observed in up to 32 different combinations of variations in various factors.

### CMU MoBo: Gross & Shi (2001)

The treadmill in the CMU 3D room was used by 25 people, who were represented in the database. The individuals engaged in four distinct walking patterns: slow walking, fast walking, walking on an incline, and walking while holding a ball. The entire set of subjects was recorded using six high-resolution color cameras evenly positioned around the treadmill.

### TUM GAID: Hofmann et al. (2014)

The TUM-GAID dataset comprises data from 305 individuals walking along two distinct indoor paths, and TUM-GAIT stands for TUM Gait from Audio, Image, and Depth. The initial trajectory proceeds from left to right, as the second trajectory progresses from right to left. These recordings were made throughout two sessions, one in January when the subjects were clothed in bulky winter boots and jackets, and the other in April when they were wearing lighter attire. These activities were recorded using a Microsoft Kinect sensor with an image resolution of 640 × 480 pixels and frame rate of 30 frames/s.

### CASIA-B: Zheng et al. (2011)

CASIA-B established in January 2005, is an extensive collection of gait information captured from 124 individuals recorded at 25 frames per second with a resolution of 320 × 240 pixels. It included 13,640 video sequences with 11 different viewpoints (ranging from 0° to 180° in 18° increments). This dataset meticulously accounts for three distinct variables: alterations in view angle, clothing, and carrying conditions. In additional, it includes human silhouettes extracted from the corresponding video files.

## Experimental results and analysis

Various state-of-the-art neural network-based algorithms have been utilized for gait recognition, showcasing different methods and impressive results in Table 5. The CNN-based model, GA-ICDNet, achieved a verification error rate of 0.67% and strong Rank-1 accuracy of 97.6%. Another approach, multiple spatiotemporal feature extraction, had an average Rank-1 accuracy of 72.9% under normal conditions. Incremental learning with covariate components resulted in an average Rank-1 accuracy of 82.2%. MSTFE exhibited robust performance with average Rank-1 accuracies of 97.6%, 94.1%, and 81.2% for normal, brisk, and casual walking, respectively. Feature fusion with a dual-path structure achieved average Rank-1 accuracies of 97.04%, 92.72%, and 80.48% under different walking conditions. Deep Convolutional Feature-based Gait Recognition surpassed 86% accuracy in challenging scenarios. Deep convolutional neural networks achieved accuracy levels above 97% on the CASIA-B dataset. GFLA and LTA demonstrated gait recognition accuracies of 97.4%, 94.5%, and 83.6% respectively, with an average rank-1 accuracy of 89.7% excluding identical-view cases on the OU-MVLP dataset with 14 probes. The Mask R-CNN and enhanced GaitSet Algorithm with the V-HPM feature mapping module (Zhang et al., 2021) displayed strong accuracy, reaching 95.26% for normal walking (NM), 89.28% for carrying a bag (BG), and 72.48% for wearing a coat cloth (CL). Leveraging the GEI generated by Mask R-CNN and CNN with Batch normalization (Inui et al., 2020) achieved over 90% accuracy when subjects carried objects/bags and 50–60% accuracy without. GaitNet, which incorporates Mask R-CNN, CNN, and LSTM (Zhang et al., 2022), achieved an exceptional identification accuracy of 99.7%. The Hybrid silhouette-skeleton-based gait representation by Cai et al. (2021), combining CNN, LSTM, and GCN, showed an average Rank-1 accuracy of 97.4% for NM, 92.1% for BG, and 81.5% for CL. The Temporal Part-based GaitPart model with Focal convolutional layer and Micro-motion Capture Module (MCM) (Fan et al., 2020) achieved an average rank-1 accuracy of 88.7% on the OU-MVLP dataset. MFINet+TSSI (Cosma & Radoi, 2020), using LSTM and ResNet, demonstrated an accuracy rate of 85% under normal walking conditions on CASIA-B. GaitGraph, using ResGCN and 2D CNN (Teepe et al., 2021), presented an average Rank-1 accuracy of 87.7% for NM, 74.8% for BG, and 66.3% for CL. Other models, such as the Koopman Operator with Fully Connected Network (Zhang et al., 2021), GaitSet Batch and Normalization Neck (Han et al., 2022), and GCN with Graph Convolution-Based Part Feature Polling (GCPFP) (Ding et al., 2022), exhibited diverse accuracies, highlighting the variety of approaches and their effectiveness in gait recognition.

**Table 5:** The method and experimental results of various state-of-the-art gait recognition techniques.

Neural network-based algorithms	Method	Experimental results	References
CNN based Model	Gate controlled and shared attention ICDNet (GA-ICDNet)	(OU-LP Bag dataset is not fine-grained labels, acted as a pre-trained model) Equal Error Rate (verification task): 0.67% Rank -1(identification task): 97.6%	Chai et al. (2021)
	Multiple spatio-temporal feature extraction	Averaged rank-1 accuracy under NM (Normal) condition: 72.9%	Su, Zhao & Li (2021)
	Integrating covariate component incremental learning with gait recognition	The overall test data for an incremental step have an average Rank-1 accuracy: 82.2%	Mu et al. (2020)
	Multi-temporal-scale feature extractor (MSTFE)	Averaged Rank-1 Accuracy: NM = 97.6% BG = 94.1% CL = 81.2%	Lin et al. (2021)
	Feature fusion with dual-path structure (local and global feature extraction) is used to build an improved cross-view gait identification system.	Averaged Rank-1 Accuracy for LT (74): NM = 97.04% BG = 92.72% CL = 80.48%	Hong et al. (2021)
	Using binary human silhouettes, deep convolutional feature-based gait recognition	Reached more than 86% in three more challenging scenarios where angle variances are significant in the cross-view experiment setting, and achieved 92% among eight out of eleven angles (using RGB photos directly without computing the gait cycles).	Işık & Ekenel (2021)
	Deep convolutional neural network	On the CASIA-B dataset, various viewing angles and environments can result in accuracy levels of above 97%.	Aung & Pluempitiwiriyawej (2020)
	GFLA and LTA	Gait recognition accuracy (%) in CASIA-B under LT (74 individuals) varied angles, settings, and situations is 97.4%, 94.5%, and 83.6%. With identical-view cases excluded, the average rank-1 accuracy for OU-MVLP (14 probes) is 89.7%.	Lin, Zhang & Yu (2021)
Mask R-CNN +CNN	Mask R-CNN and improved GaitSet algorithm with V-HPM feature mapping module	Gait Recognition Accuracy: NM = 95.26% BG = 89.28% CL = 72.48%	Zhang et al. (2021)
	Utilizing GEI produced by Mask R-CNN and CNN with reinforcement from Batch normalization	Achieved more than 90% of accuracy (with subject carrying object/bag) and 50% to 60% of accuracy (without subject carrying object/bag)	Inui et al. (2020)
Mask R-CNN+CNN+LSTM	GaitNet (autoencoder + LSTM)	Identification Accuracy: 99.7%	Zhang et al. (2022)
CNN+LSTM+GCN	Hybrid silhouette-skeleton-based gait representation (FPFE and MCM)	Average Rank-1 Accuracy: NM = 97.4% BG = 92.1% CL = 81.5%	Cai et al. (2021)
Fconv	Temporal part-based GaitPart model (focal convolutional layer and micro-motion capture module-MCM)	On the OU-MVLP, the average rank-1 accuracy is 88.7%.	Fan et al. (2020)
LSTM+ResNet	MFINet and TSSI	Accuracy Rate 85% Accuracy results of normal walking condition on CASIA-B: MFINet - concat = 85.81% MFINet - sum = 85.09% Accuracy results of clothing variations condition on CASIA-B: MFINet - concat = 11.72% MFINet - sum = 14.29% Accuracy results of different carrying conditions on CASIA-B: MFINet - concat = 18.74% MFINet - sum = 19.32%	Cosma & Radoi (2020)
ResGCN+2D CNN	GaitGraph uses graph convolutional network (GCN) and skeletal poses to provide a cutting-edge model-based technique for gait identification.	Averaged Rank-1 accuracy: NM = 87.7% BG = 74.8% CL = 66.3%	Teepe et al. (2021)
Fully connected network	Koopman operator	With identical-view cases excluded, the average rank-1 accuracy for OU-MVLP = 74.7%.	Zhang et al. (2021)
	GaitSet batch and normalization neck	With identical-view cases excluded, the average rank-1 accuracy for CASIA-B = 87.5%.	Han et al. (2022)
GCN	This approach utilizes GaitSet as its core framework and addresses the challenge of feature misalignment by incorporating a technique known as graph convolution-based part feature polling (GCPFP).	With identical-view cases excluded, the average rank-1 accuracy for CASIA-B = 62.4%.	Ding et al. (2022)

Table 6 provides a comprehensive comparative analysis of various gait recognition models, assessing their performance across different walking states (0° to 180°). Each model was evaluated based on the mean accuracy, along with specific recognition rates for normal walking (NM), carrying a bag (BG), and wearing a coat cloth (CL). Table 6, highlights the remarkable results for each degree of NM (normal walking), BG (walking with a bag), and CL (walking with clothing variations). For example, at 0°, the Multi-Temporal-Scale Feature Extractor yielded valuable results in NM and BG with accuracies of 96.7% and 93.7%, respectively, as published by Lin et al. (2021). Additionally, the Spatio-Temporal Representation Model proposed by Liu et al. (2021) showed good performance in CL with an accuracy of 76.7%. Likewise, the remarkable results for all 11 degrees in NM, BG, and CL are highlighted.

**Table 6:** Accuracy (%) of gait recognition under different angles, settings, and conditions excluding identical-view cases in CASIA-B. Bold values refers to the best result of different models.

Method used	Walking states	0°	18°	36°	54°	72°	90°	108°	126°	144°	162°	180°	Mean	References
Multiple spatio-temporal feature extraction	NM	93.3	97.1	98.6	97.1	93.6	91.5	95.2	97.7	99.0	97.0	89.0	95.4	Su, Zhao & Li (2021)
	BG	84.4	91.0	93.3	92.2	83.6	80.0	83.6	91.7	95.1	92.3	81.7	88.1	
	CL	64.9	78.6	81.3	78.1	75.0	71.1	74.2	76.0	76.3	75.4	59.0	73.6	
Mask R-CNN and improved GaitSet algorithm with V-HPM feature mapping module	NM	92.9	97.4	99.0	97.3	93.0	91.7	94.8	98.0	98.6	97.3	87.8	95.3	Zhang et al. (2021)
	BG	86.0	91.6	93.3	90.8	88.1	82.5	86.4	92.8	94.8	92.5	83.2	89.3	
	CL	65.4	79.4	82.6	77.0	72.2	68.5	72.3	75.4	74.4	72.9	57.2	72.5	
Spatio-temporal representation model	NM SG-6	90.4	93.8	96.8	96.7	92.0	92.2	92.2	94.6	96.8	94.8	88.6	93.5	Liu et al. (2021)
	BG SG-6	90.6	91.9	94.1	91.2	87.9	84.5	86.4	90.6	90.6	93.3	90.0	90.1	
	CL SG-6	**76.7**	82.7	88.3	84.2	80.4	78.5	**84.6**	83.9	83.5	80.6	**71.5**	81.3	
GaitNet (autoencoder + LSTM + loss function)	NM	93.1	92.6	90.8	92.4	87.6	95.1	94.2	95.8	92.6	90.4	90.2	92.3	Zhang et al. (2022)
	BG	88.8	88.7	88.7	94.3	85.4	92.7	91.1	92.6	84.9	84.4	86.7	88.9	
	CL	50.1	60.7	72.4	72.1	74.6	78.4	70.3	68.2	53.5	44.1	40.8	62.3	
Hybrid silhouette FPFE and MCM	NM	96.4	98.7	**99.8**	**98.9**	96.1	94.1	96.6	**98.9**	**99.8**	**99.1**	93.2	97.4	Cai et al. (2021)
	BG	89.1	96.0	94.9	93.8	89.4	85.8	91.1	94.8	95.6	94.4	88.1	92.1	
	CL	75.4	88.2	89.1	86.2	79.0	75.0	79.7	84.0	84.3	**85.2**	71.2	81.5	
Temporal part-based GaitPart model	NM #5-6	94.1	98.6	99.3	98.5	94.0	92.3	95.9	98.4	99.2	97.8	90.4	96.2	Fan et al. (2020)
	BG #1-2	89.1	94.8	96.7	95.1	88.3	**94.9**	89.0	93.5	96.1	93.8	85.8	91.5	
	CL #1-2	70.7	85.5	86.9	83.3	77.1	72.5	76.9	82.2	83.8	80.2	66.5	78.7	
Multi-temporal-scale feature extractor	NM #5-6	**96.7**	**99.0**	99.1	97.9	96.6	**95.5**	**97.6**	**98.9**	99.1	98.7	**94.3**	**97.6**	Lin et al. (2021)
	BG #1-2	**93.7**	**96.6**	96.9	94.7	92.8	88.8	91.8	95.6	98.0	**97.0**	90.0	94.1	
	CL #1-2	73.5	86.9	88.6	85.1	80.8	76.4	80.4	83.7	85.8	83.7	69.0	81.2	
Cross view recognition model	NM - LT(74)	95.1	98.9	99.6	98.3	96.2	93.7	96.3	98.8	99.6	98.6	92.5	97.0	Hong et al. (2021)
	BG - LT(74)	90.7	96.1	**97.5**	**95.8**	89.2	84.4	90.3	94.8	97.9	94.6	88.6	92.7	
	CL - LT(74)	75.6	84.9	89.3	84.0	80.8	76.2	76.8	83.3	83.6	82.7	68.2	80.5	
GFLA and LTA	NM - LT(74)	96.0	98.3	99.0	97.9	**96.9**	95.4	97.0	**98.9**	99.3	98.8	94.0	97.4	Lin, Zhang & Yu (2021)
	BG - LT(74)	92.6	96.6	96.8	95.5	**93.5**	89.3	**92.2**	**96.5**	**98.2**	96.9	**91.5**	**94.5**	
	CL - LT(74)	76.6	**90.0**	**90.3**	**87.1**	**84.5**	**79.0**	84.1	**87.0**	**87.3**	84.4	69.5	**83.6**	
ResGCN and 2D CNN	NM #5-6	85.3	88.5	91.0	92.5	87.2	86.5	88.4	89.2	87.9	85.9	81.9	87.7	Teepe et al. (2021)
	BG #1-2	75.8	76.7	75.9	76.1	71.4	73.9	78.0	74.7	75.4	75.4	69.2	74.8	
	CL #1-2	69.6	66.1	68.8	67.2	64.5	62.0	69.5	65.6	65.7	66.1	64.3	66.3	

Multiple spatio-temporal feature extraction (Su, Zhao & Li, 2021) demonstrate high recognition rates, especially for NM and BG, achieving a mean accuracy of 95.4%. Mask R-CNN and the improved GaitSet Algorithm (Zhang et al., 2021) showed strong overall performance with a mean accuracy of 95.3%, which is particularly effective for NM and BG. The spatio-temporal representation model by Liu et al. (2021) yields competitive results, securing a mean accuracy of 93.5%, which is particularly effective for NM and BG. GaitNet (Autoencoder + LSTM + Loss function) (Zhang et al., 2022) exhibit varying performance across walking states, with a mean accuracy of 62.3%, showing particular strength in NM and BG. Hybrid silhouette FPFE and (Cai et al., 2021) performed well, with a mean accuracy of 81.5%, which is especially effective for NM and BG. The Temporal Part-based GaitPart model (Fan et al., 2020) demonstrates good recognition rates, particularly for NM and BG, with a mean accuracy of 78.7%. The Multi-Temporal-Scale Feature Extractor (Lin et al., 2021) showcases strong overall performance with a mean accuracy of 97.6%, particularly effective for NM and BG. The Cross-view recognition model (Hong et al., 2021) achieves high recognition rates across walking states, with a mean accuracy of 97.0%, particularly effective for NM and BG. GFLA and LTA (Lin et al., 2021) exhibited a strong recognition performance with a mean accuracy of 97.4%, which is particularly effective for NM and BG. ResGCN and 2D CNN (Teepe et al., 2021) exhibited varying performances across walking states, with a mean accuracy of 87.7%, showing particular strength in NM and BG.

**Figure 6:**
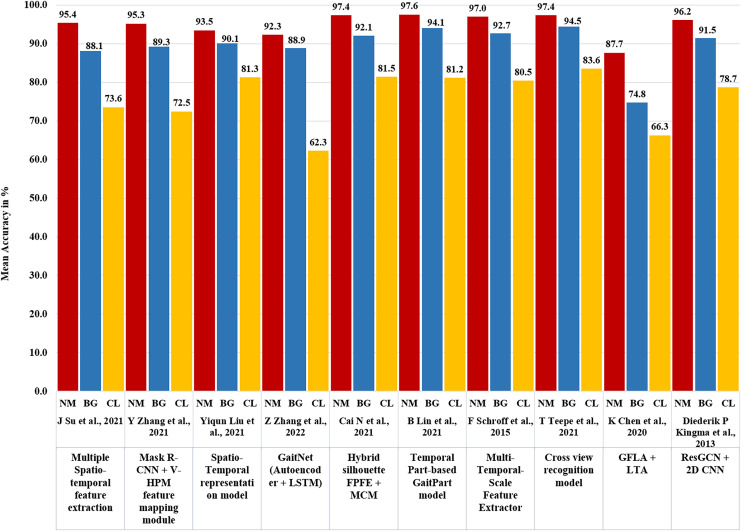
Mean accuracy of the different walking conditions (NM, BG and CL) across various techniques.

Figure 6 represents the mean walking states across various studies as referenced. Walking states are categorized into three conditions: normal (NM), carrying a bag (BG), and wearing a coat cloth (CL). Each reference (indicated by the reference number) shows the mean values of various studies, with NM generally having the highest mean value, followed by BG, and CL.

## Conclusions

This survey explores extensive applications and technological advancements in gait recognition, an emerging field in biometrics. Gait recognition, which analyzes distinctive walking patterns, offers unique advantages such as long-distance capture, non-cooperation requirement, and difficulty in imitation, making it a reliable identification method applicable in various domains including criminal investigation, healthcare, sports, security, and robotics.

This research highlights the significance of gait recognition in criminal investigations, where it can serve to cross-check alibis, aid in criminal profiling, act as forensic evidence, and enhance witness testimonies. Moreover, the technology finds utility in healthcare for rehabilitation monitoring and fall detection, in sports for performance optimization and injury prevention, and in security for biometric identification and surveillance.

The article meticulously examines different approaches for human identification using gait recognition technology, including appearance-based methods, model-based methods, sensor-based methods, deep learning methods, fusion methods, 3D gait recognition, and spatiotemporal methods, each with its advantages and applications. Notably, the integration of deep learning has revolutionized gait identification by addressing challenges such as automatic feature learning, robustness to variations, and transfer learning, leading to remarkable accuracy and real-world practicality.

Moreover, this article outlines the general steps involved in human gait recognition systems utilizing deep learning techniques, encompassing data acquisition, preprocessing, feature extraction, model training, recognition, and post-processing. The research discusses various neural network-based algorithms and experimental results, showcasing the effectiveness of different approaches in accurately identifying individuals under diverse conditions and angles. Noteworthy results include high accuracy rates achieved by hybrid approaches combining silhouette-skeleton-based representations with deep learning techniques, as well as the integration of sophisticated models such as Mask R-CNN, LSTM, ResNet, and GCN, which have significantly advanced the field.

Despite challenges such as sensitivity to environmental conditions, limited data availability, and computational intensity, ongoing research focuses on refining techniques, improving accuracy, and adapting to evolving gait patterns, thus ensuring gait recognition remains a dynamic and promising avenue in biometrics and security.

This study provides a comprehensive analysis of recent advancements in gait recognition using deep learning techniques and consolidates the latest findings and results from various gait recognition models, offering a holistic view of recent advancements in the field. To strengthen this article, it includes a detailed comparative review of experimental analyses from the existing literature to offer unique insights into previous work, along with discussing the advantages, limitations, methods, datasets, and significant results in the field. Additionally, we focus on developments after 2020 to guide researchers and readers, with the potential for future extensions. The mean accuracy values reported in recent studies ranged from 62.3% to 97.6%, highlighting the variability in performance across different conditions and datasets. Most of the gait recognition approaches used OU-MVLP, OU-LP Bag, OU-Lp Bag-β, OU-ISIR, Front-View Gait (FVG), USF, CMU MoBo, TUM GAID and CASIA-B as their datasets. Among these CASIA -B yielded desirable results and was used more frequently across different models. The Multi-Scale Temporal Feature Extractor (MSTFE) model was proposed by Lin et al. (2021) gave a better mean accuracy in NM of 97.6% when compared to other models, followed by the (GFLA and LTA) method implemented by Lin, Zhang & Yu (2021) yielding a good mean accuracy value in BG and CL of 94.5% and 83.6%, respectively. These findings underscore the varied strengths of different gait recognition models, emphasizing the importance of selecting models based on specific application requirements, and the need to recognize different walking states.

Future research could focus on improving the accuracy of gait recognition models across diverse environments, lighting conditions, and clothing variations, by utilizing real-world datasets to validate these improvements. Furthermore, formulating methods to ensure that factors such as clothing variations, bags, or other carried items do not affect the identification of individuals would be crucial. Developing anti-spoofing techniques to detect and prevent attacks on gait recognition systems enhancing their security and robustness holds significant research potential.

## Additional Information and Declarations

### Data Availability

The following information was supplied regarding data availability: This is a literature review.
